# Protein-Based Electrospun Nanofibers Doped with Selenium Nanoparticles for Wound Repair

**DOI:** 10.3390/pharmaceutics17101276

**Published:** 2025-09-30

**Authors:** Marco Ruggeri, Simone Marsani, Amedeo Ungolo, Barbara Vigani, Eleonora Bianchi, Cèsar Viseras, Silvia Rossi, Giuseppina Sandri

**Affiliations:** 1 Department of Drug Sciences, University of Pavia, Viale Taramelli 12, 27100 Pavia, Italy; 2 Department of Pharmacy and Pharmaceutical Technology, University of Granada, Campus of Cartuja, 18071 Granada, Spain

**Keywords:** chronic wounds, gliadin, gelatin, selenium nanoparticles, antioxidant properties, wound healing

## Abstract

**Background/Objectives**: The design of scaffolds that mimic the extracellular matrix has gained increasing attention in regenerative medicine. This study aims to develop and characterize electrospun nanofibrous scaffolds based on pullulan blended with either gelatin or gliadin and doped with selenium nanoparticles (Se NPs), to assess the influence of protein type and Se NP doping on scaffold performance and regenerative potential. **Methods**: Se NPs were synthesized via redox reaction and stabilized using pullulan. Electrospun scaffolds were then prepared by blending pullulan-stabilized Se NPs with either gelatin or gliadin. The resulting fibers were characterized using a multidisciplinary approach, including physicochemical (morphology, fiber dimension, swelling capacity, surface zeta potential, mechanical properties) and preclinical properties (antioxidant properties, fibroblast adhesion and proliferation, collagen expression). **Results:** Protein type influenced fiber morphology and dimensions, as well as mechanical behavior, with gelatin-based scaffolds demonstrating smaller fiber diameters and higher mechanical properties. The doping with Se NPs enhanced scaffold antioxidant properties without affecting fiber formation. Moreover, all scaffolds supported fibroblast proliferation, but those containing Se NPs showed enhanced modulation of ECM gene expression. **Conclusions**: The results show that scaffolds doped with Se NPs exhibited superior performance compared to the undoped counterparts, offering promising platforms for chronic wound reparation.

## 1. Introduction

Chronic wounds, such as diabetic ulcers or pressure sores, may require several years to heal, leading to an increased risk of recurrent infections, amputations, and systemic complications, significantly impacting both the patient’s quality of life and healthcare costs [1]. In this context, research is focusing on the development of porous three-dimensional scaffolds capable of mimicking the extracellular matrix (ECM) and promoting a microenvironment suitable for cell adhesion, proliferation, and tissue regeneration [2,3].

The choice of the polymer matrix plays a crucial role for determining the mechanical and physicochemical properties of the final scaffold, as well as for guiding cell–material interactions and biological responses [4,5]. Among natural biomaterials, proteins have gained increasing attention thanks to their intrinsic bioactivity and structural similarity to native ECM components. Gelatin, a collagen-derived protein obtained from animal connective tissues, is widely used in biomedical applications due to its excellent biocompatibility, biodegradability, and ability to support cell adhesion and proliferation through integrin-binding domains such as RGD motifs [6,7,8,9]. However, being an animal-derived product, gelatin raises concerns in terms of sustainability, ethical sourcing, and risk of immunogenicity or zoonotic contamination, which has led to the exploration of plant-based alternatives [10,11]. Although less explored than gelatin in tissue engineering, gliadin represents an interesting plant-based alternative for scaffold production, especially when the exclusion of animal-derived materials is required. Gliadin, a wheat-derived storage protein, is amphiphilic due to distinct hydrophilic and hydrophobic domains, and characterized by antioxidant and mucoadhesive properties [12,13].

Innovative strategies in wound healing have focused on designing novel systems that can provide an optimal microenvironment within the wound, specifically addressing oxidative stress and inflammation. Studies have designed several platforms capable of releasing antioxidants or anti-inflammatory agents directly at the wound site, leading to faster healing and reduced oxidative damage [14,15]. These studies pointed out that doping bioactive components into scaffolds can generate multifunctional systems capable of offering structural support and, at the same time, actively modulating the wound microenvironment, supporting the natural progression of the healing process. A promising strategy involves the doping of selenium nanoparticles (Se NPs) into scaffolds due to their antioxidant, anti-inflammatory, and antimicrobial properties [16]. Selenium, an essential immunonutrient, exerts its functions in the human body through selenoproteins, which play key roles in thyroid hormone regulation, immune modulation, and antioxidant defense [17,18]. During wound healing, selenoproteins such as glutathione peroxidase (GPX)-1, GPX-4, and selenoprotein P, contribute to inhibition of inflammatory cytokines and elimination of super radical ions in the inflammatory phase [19,20]. Several studies have reported that the beneficial or toxic effects of selenium are strongly influenced by its chemistry [21]. In particular, orange-red zero-valent Se NPs, obtained via redox reactions, are characterized by lower toxicity than other selenium species [22]. However, their clinical application is hindered by poor colloidal stability, as they are prone to aggregation, forming less active gray or black elemental selenium [23]. To address this issue, stabilizing agents are commonly employed in order to prevent aggregation and guarantee their properties and effectiveness [21,24].

Given these premises, the aim of this work was the design and the development of gliadin-based scaffolds doped with Se NPs to repair chronic wounds and accelerate wound healing. Gliadin performance was compared with gelatin, a well-known component of scaffolds for wound healing. Firstly, Se NPs were synthesized using pullulan to maintain their colloidal stability. Subsequently, gelatin or gliadin was incorporated into the polymeric blends and electrospun to produce nanofibrous scaffolds. Particular attention was given to the influence of the protein origin and the presence of SeNPs on fiber morphology and size, swelling capacity, surface zeta potential, mechanical properties, and antioxidant performance. Moreover, the scaffolds were evaluated for their ability to support fibroblast adhesion and proliferation, as well as their modulation of ECM-related gene expression.

## 2. Materials and Methods

### 2.1. Materials

For Se NP synthesis, the following reagents were used: sodium selenite (Na_2_SeO_3_, 99%, Sigma-Aldrich, Milan, Italy), L-ascorbic acid (99%, Sigma-Aldrich, Milan, Italy), and pullulan (MW 200–300 kDa, food grade, Hayashibara, Japan, Giusto Faravelli, Milan, Italy). For the scaffold preparation, the following proteins were used: gelatin (from bovine skin, gel strength ~225 g Bloom, Type B, water solubility 50 mg/mL), gliadin from wheat (Sigma-Aldrich, Italy). Citric acid (99%, Sigma-Aldrich, Italy) was used as crosslinking agent.

### 2.2. Methods

#### 2.2.1. Synthesis and Characterization of Selenium Nanoparticles

Se NPs were synthesized by reducing Na_2_SeO_3_ in the presence of pullulan as a stabilizing agent. Two different solvent systems were used: water and a 30% *v*/*v* ethanol/water mixture. In both cases, a 1:500 molar ratio of Na_2_SeO_3_ to pullulan was used, with pullulan dissolved to a final concentration of 15% *w*/*v*. The polymer solutions were magnetically stirred for 3 h to ensure complete dissolution. Separately, a 10-fold molar excess of ascorbic acid (used as reducing agent) relative to Na_2_SeO_3_ was dissolved in the corresponding solvent (water or 30% *v*/*v* ethanol). The reduction reaction was initiated by adding the 3 mL ascorbic acid solution dropwise to 30 mL pullulan/Na_2_SeO_3_ solution under high-shear homogenization (Ultra-Turrax T25, IKA-Werke GmbH, Staufen im Breisgau, Germany) at 25,000 rpm for 27 min.

The resulting nanoparticle suspension was characterized by means of a transmission electron microscopy (TEM, Jeol JEM-1200 EX II, Tokyo, Japan), while particle size distribution and ζ-potential were assessed using dynamic light scattering (Litesizer 500, Anton Paar, Turin, Italy).

XRPD analysis of Se NPs was carried out by means of a X’Pert Pro model (Malven Panalytical, Madrid, Spain) in the range 4–75° 2θ.

#### 2.2.2. Preparation of Electrospun Fibers

Gelatin or gliadin (10% *w*/*v*) was added to the aqueous or hydroalcoholic selenium nanoparticle (Se NP) solutions and stirred until complete dissolution. Citric acid (10% *w*/*v*) was then added as a crosslinking agent. Two blank solutions were also prepared, containing pullulan with either gelatin or gliadin, in the absence of SeNPs. Nanofibers were prepared via electrospinning using a STKIT-40 setup (Linari Engineering, Pisa, Italy), under the following conditions: applied voltage 20 kV, spinneret/collector distance 17 cm, flow rate 0.86 mL/h, inner diameter 0.8 mm, relative humidity 35% at 25 °C for gliadin-based blends and 15% at 40 °C for gelatin-based blends, and an electrospinning duration of 2 h. Subsequently, the scaffolds were crosslinked at 180 °C for 2 h. The theoretical Se% on dry scaffolds was 0.04% *w*/*w*.

#### 2.2.3. Physicochemical Characterization

The morphology of the electrospun scaffolds was assessed by means of scanning electron microscopy (SEM Phenom Pure G6 Desktop, Thermo Fisher Scientific, Alfatest, Cernusco sul Naviglio, Milan, Italy) using the following parameters: voltage 20 kV, working distance 5.8 mm, detector SED. Samples were mounted on metal stubs using double-side adhesive carbon tape and sputtered with a thin layer of gold under vacuum. Nanofiber diameters were estimated using image analysis software (Image J, version 1.53, National Institutes of Health, Bethesda, MD, USA).

The presence and distribution of selenium nanoparticles within the polymer matrix were investigated using TEM (Jeol JEM-1200 EX II, Tokyo, Japan), performed on fibers that were directly electrospun onto copper grids.

Thermogravimetric analysis (TGA) was evaluated using a TGA instrument (Mettler-Toledo GmbH, Madrid, Spain) [25]. Samples (~20 mg) were weighed into aluminum pans and heated from 25 °C to 800 °C at a rate of 10 °C/min under atmospheric air flow.

FTIR spectra were acquired using a JASCO 6200 spectrometer (JASCO Deutschland GmbH, Pfungstadt, Germany) equipped with a germanium ATR probe. Measurements were carried out in the range of 400–4000 cm^−1^ with a resolution of 2 cm^−1^.

#### 2.2.4. Swelling Properties and Contact Angle

The swelling behavior of the electrospun scaffolds was assessed by incubating exact amount of the samples in phosphate-buffered saline (PBS, pH 7.4) at 37 °C under static conditions. At fixed time points (1, 3, and 6 days), the samples were withdrawn, gently blotted to remove excess liquid, and weighed. The swelling index (SI) was calculated using the following equation:

$$SI=\frac{W_{t}-W_{0}}{W_{0}}$$

where *W*_0_ is the initial dry weight and *Wₜ* is the weight at each time point.

Moreover, SEM analysis was carried out after 6 days of hydration to visualize morphological changes in the fiber structure on dried scaffolds.

Contact angle measurements were performed by the sessile drop method using a DMe-211 Plus instrument (FAMAS software version 3.3, Kyowa, Osaka, Japan). The droplet was imaged after 100 s on the scaffold, and its contact angle was measured.

#### 2.2.5. Surface Zeta Potential

The surface zeta potential of the electrospun scaffolds was measured using a SurPASS electrokinetic analyzer (Anton Paar, Turin, Italy) equipped with the cylindrical cell. Samples were cut into circular disks (1 cm diameter) and mounted in the measuring cell [15]. Measurements were performed using 1 mM KCl solution over a pH range of 2 to 9. The surface zeta potential and the isoelectric point (IEP) were measured.

#### 2.2.6. Mechanical Properties

The mechanical properties of the nanofibrous scaffolds were evaluated using a texture analyzer (TA-XT Plus, Stable Microsystems, Venice, Italy) equipped with a 5 kg load cell. Rectangular samples (30 mm × 10 mm, 0.2 mm thickness) were prepared and mounted between two tensile grips, with an initial grip distance of 20 mm. The upper grip was then moved at a constant rate of 5.0 mm/s until the sample ruptured. Mechanical performance was assessed under both dry and hydrated conditions, and the results were expressed as maximum force, elongation at break, and Young’s modulus.

#### 2.2.7. Antioxidant Activity Evaluation

The antioxidant activity of the electrospun scaffolds was assessed by monitoring their radical scavenging activity (RSA%) using a 2,2-diphenyl-1-picrylhydrazyl (DPPH) assay. Then, 10 mg of fibers were incubated in 2 mL of PBS at 37 °C, and, at fixed time points (1, 3, 6, 9, and 13 days) the supernatants were collected for analysis. The collected supernatants were mixed 1:1 *v*/*v* with a DPPH solution (300 μg/mL in methanol) in a 96-well plate, and control wells were prepared by replacing the supernatant with water. The plate was incubated in the dark at room temperature for 30 min. Subsequently, the absorbance was measured at 597 nm using a microplate reader (FLUOstar^®^ Omega, BMG LABTECH, Aylesbury, UK) and the RSA% was calculated [26].

#### 2.2.8. In Vitro Testing

Circular scaffolds (area = 0.7 cm^2^) were placed in the bottom of a 48-well plate, and 35,000 normal human dermal fibroblasts (NHDFs from juvenile foreskin, PromoCell, Sigma-Aldrich, Milan, Italy) were seeded onto each membrane and incubated for 3 and 6 days. At both time points, cell proliferation was evaluated using the AlamarBlue^®^ assay. After removing the medium, wells were washed and 300 μL of AlamarBlue^®^ solution (10% *v*/*v* in phenol red-free DMEM) was added to each well. Plates were incubated at 37 °C for 3 h in the dark, and the fluorescence intensities were read using a microplate reader (FLUOstar^®^ Omega, BMG LABTECH, Aylesbury, UK) at ex/em 560/590 nm.

Moreover, after 6 days of growth, samples were fixed with 3% glutaraldehyde (Sigma-Aldrich, Italy) for 1 h at 4 °C, then washed with PBS. Samples were incubated for 40 min in the dark with 100 μL of Phalloidin-FITC solution (50 μg/mL) to stain the cytoskeleton, followed by PBS washes. Nuclei were stained with Hoechst 33,258 solution (1:10,000 dilution in PBS) for 10 min in the dark and washed again. Samples were mounted on microscope slides and visualized using a confocal laser scanning microscope (CLSM, Leica TCS SP2, Leica Microsystems, Wetzlar). The cell density was calculated using an image analysis software (Image J, version 1.53, National Institutes of Health, Bethesda, MD, USA). In parallel, after 6 days of culture, total RNA was extracted from NHDFs grown onto the membranes using TriZol agent (ThermoFisher, Milan, Italy). Quantitative real-time PCR (qRT-PCR) was carried out to assess the expression of COL1A1 using the SsoAdvanced Universal SYBR Green Supermix (Bio-Rad Laboratories Srl, Segrate, Milan, Italy). GAPDH was employed as the housekeeping gene for normalization of gene expression levels. The thermal cycling protocol was performed as previously described [27]: initial polymerase activation at 95 °C for 30 s, followed by 39 cycles of DNA denaturation at 95 °C for 15 s and annealing at 60 °C for 30 s.

## 3. Results and Discussion

### 3.1. Se NP Synthesis and Characterization

**Figure 1:**
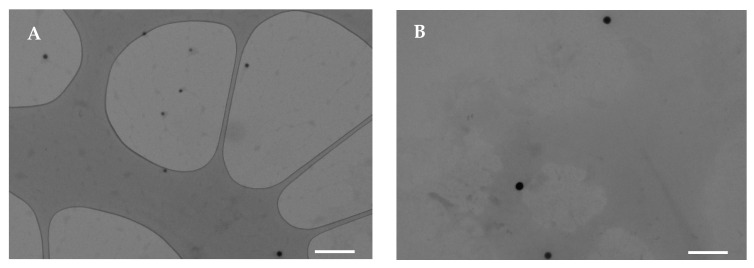
TEM micrographs of Se NPs prepared in water (**A**) or in ethanol/water mixture (**B**) (white line–scale bar: 500 nm).

Se NPs are commonly stabilized using biocompatible agents, such as polysaccharides, proteins, or *polyphenols*, due to their inherent instability and limited biological activity [28]. In this work, pullulan was chosen as the stabilizer and dispersing agent for Se NP synthesis due to its spinnability for the subsequent electrospinning process. Pullulan-stabilized Se NPs were synthesized by reacting sodium selenite with ascorbic acid, which is characterized by biocompatibility and low toxicity compared to other reducing agents. The gradual transition of the transparent sodium selenite solution to a reddish color confirmed the successful formation of Se NPs [29]. The nanoparticles were prepared in two different media, pure water and a water/ethanol mixture, in order to subsequently add gelatin and gliadin, respectively. Figure 1 reports TEM micrographs of Se NPs stabilized with pullulan, prepared both in water and in a water/ethanol mixture. Both Se NPs appear as spherical dark spots, with no aggregation, suggesting that pullulan effectively prevents particle coalescence during synthesis. On the other hand, in the absence of a stabilizing agent, Se NPs aggregated rapidly therefore, pullulan plays a crucial role in maintaining colloidal stability.

**Figure 2:**
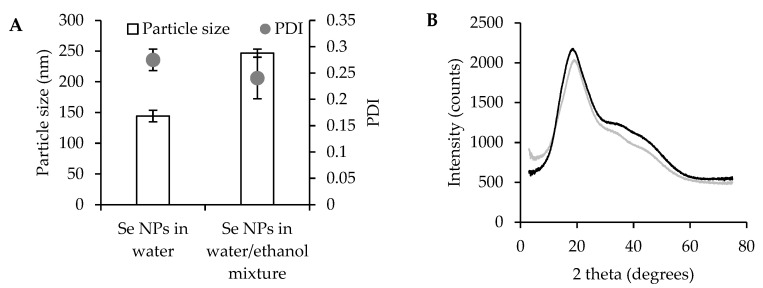
Particle size and PDI (**A**) of Se NPs prepared in water or in ethanol/water mixture, mean values ± s.d.; n = 3. One-way ANOVA; Scheffé test (*p* ≤ 0.05), particle size: Se NPs in water vs. Se NPs in water/ethanol mixture. XRPD spectra (**B**) of Se NPs prepared in water (black line) or in ethanol/water mixture (gray line).

Moreover, the DLS results (Figure 2A, together with their size distribution reported in Figure S1) highlight that Se NPs in water exhibit a smaller average hydrodynamic diameter (140 nm) compared to those prepared in a water/ethanol mixture (240 nm). The presence of ethanol conceivably interferes with the process of self-assembly or nucleation, leading to larger, polydisperse particles, although pullulan avoids aggregation. In both cases, the PDI was about 0.25, indicating a mid-range polydisperse size distribution. In addition, the mean particle diameters observed by TEM are about 80 nm for Se NPs prepared in water and 140 nm for those obtained in the water/ethanol mixture, values lower than the corresponding hydrodynamic diameters detected by DLS. This deviation can be related to the two different techniques: TEM reflects the size of dry nanoparticles, while the slightly larger hydrodynamic diameters detected by DLS are presumably due to solvation layers and possible weak interparticle interactions in the dispersion. However, there is no clear evidence of aggregation, indicating that colloidal stability was maintained and that large aggregates were avoided, supporting more predictable biological interactions.

XRD spectra of pullulan-stabilized Se NPs show a broad diffraction peak at around 20 2θ (Figure 2B), indicating that the selenium nanoparticles adopt an amorphous state. According to the literature, elemental selenium exists in various allotropic forms, including two amorphous phases (red and black) [30]. In this work, the synthesized Se NPs exhibit a stable red coloration, typical of amorphous red selenium. It is conceivable that pullulan plays a crucial role in stabilizing the Se solid state, preventing aggregation and maintaining the colloidal stability. Similar results have been reported in previous studies, highlighting the amorphous nature of Se NPs when stabilized by β-lactoglobulin, ferulic acid, and other polysaccharides [21,28,31,32].

### 3.2. Electrospun Scaffold Production

Gelatin and gliadin were added to the aqueous and hydroalcoholic Se NP suspensions, respectively. The resulting polymeric blends were then subjected to the electrospinning process in order to obtain fibrous scaffolds. Each blend, either undoped and doped with Se NP, was stable during the entire electrospinning process, with continuous jet formation and no signs of needle clogging or interruptions.

**Figure 3:**
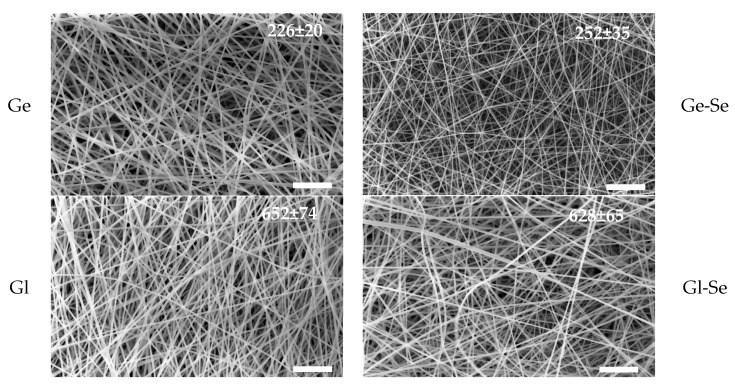
SEM micrographs of scaffolds: Ge, Ge-Se, Gl, Gl-Se (while line–scale bar: 10 μm). In the insets, the mean diameters of fibers (nm) are reported (mean values ± s.d., n = 30).

Figure 3 shows SEM images of electrospun fibers together with the mean fiber diameters, while the diameter distribution is reported in Figure S2. The electrospun fibers exhibit homogeneous morphology, with uniform diameters, smooth surfaces, and regular structures. The doping with Se NPs does not appear to alter the fiber structure and morphology. Moreover, independently from the Se NP doping, gelatin-based fibers are characterized by diameters ranging from approximately 220 to 250 nm, while gliadin-based fibers are significantly thicker, with diameters of about 620–650 nm. This difference can be related to the inherent physicochemical properties of the two proteins. Gliadin, being more hydrophobic, forms polymeric blends of higher viscosity compared to gelatin at equivalent concentrations. During electrospinning, solution viscosity is a key parameter influencing jet elongation and fiber thinning; therefore, more viscous gliadin blends lead to thicker fibers, as shown in Figure S3 [33].

**Figure 4:**
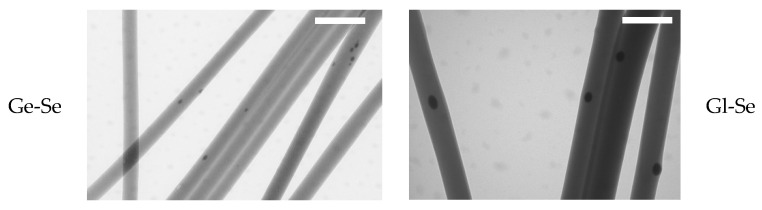
TEM micrographs of Se NP-doped electrospun fibers (scale bar 500 nm), showing Se NP doping in the individual fibers.

Moreover, the presence of Se NPs within the electrospun fibers is demonstrated by TEM analysis (Figure 4). The nanoparticles are clearly visible as dark and electron-dense spots embedded within the polysaccharide/protein matrix. This proves that the electrospun nanofibers are successfully doped with Se NPs, although a non-uniform distribution along the individual fibers can be observed.

### 3.3. Physicochemical Characterization

**Figure 5:**
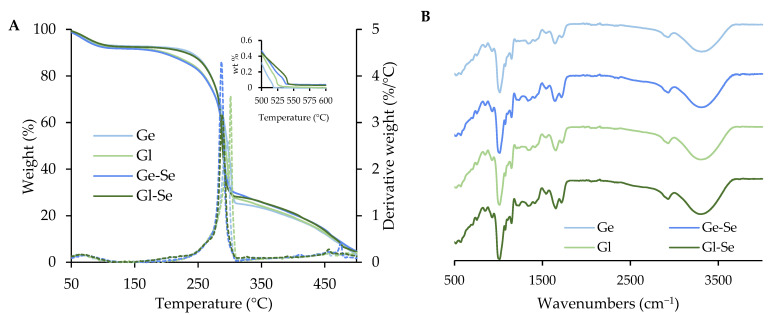
Thermal analysis (TGA and DTG, (**A**)) and FTIR spectra (**B**) of electrospun scaffolds. The inset in the left panel shows a magnified view of the 500–600 °C range.

The thermogravimetric analysis of the electrospun fibers (Ge, Ge-Se, Gl, Gl-Se) is reported in Figure 5A. The thermograms reveal three stages of degradation. The first weight loss of approximately 10% occurs between 40 °C and 110 °C and is related to the evaporation of adsorbed and bound water (peak temperature: 75–85 °C). The second stage represents the main degradation event, which begins around 160–170 °C and peaks at about 300 °C (peak temperature: 285–300 °C). During this stage, the scaffolds lose about 75% of their mass due to the thermal decomposition of the organic matrix, resulting from cleavage of peptide bonds, degradation of side chains, and the release of volatile low molecular weight fragments. The third stage, starting at around 300 °C and extending up to 520–550 °C (peak temperature: 450–475 °C), involves the degradation of more stable carbonaceous residues. This phase leads to the complete decomposition of organic matter in the undoped scaffolds. However, in the Se NP-doped scaffolds (Ge-Se and Gl-Se), a residual mass of about 0.04% can be appreciated and persists beyond 550 °C. As this residue is not present in the undoped samples, it can be attributed to the inorganic selenium component, and this value corresponds to the theoretical selenium content.

The FTIR spectra of the electrospun fibers (Figure 5B) are dominated by the characteristic absorption bands of pullulan, which represents the main component of the fibers in dry weight. In particular, all spectra show a broad band at about 3300 cm^−1^ related to OH stretching and a signal near 2900 cm^−1^ corresponding to the CH bond [34,35]. However, although the signals are partially overlapped by the pullulan background, the presence of proteins is suggested by bands at around 1650 cm^−1^ and 1540 cm^−1^, which are related to amide I and amide II, respectively [36,37]. On the other hand, scaffold doping with Se NPs does not lead to the formation of new chemical bonds, as largely expected. This is conceivably due to the low content of Se NPs within the polymeric matrix of the scaffold, as confirmed by TGA analyses.

XPRD analyses of Ge, Ge-Se, GI, and GI-Se were also performed. However, all samples exhibited amorphous profiles, with no detectable peaks, due to the dominant signal contribution of pullulan, which is the major component of the systems.

### 3.4. Swelling Index and Contact Angle

The hydration properties of the scaffolds are essential for skin tissue regeneration, as they should promote hemostasis and maintain a balance between exudate absorption and avoidance of wound bed dehydration, particularly during the proliferative or granulation phase of healing [38,39].

**Figure 6:**
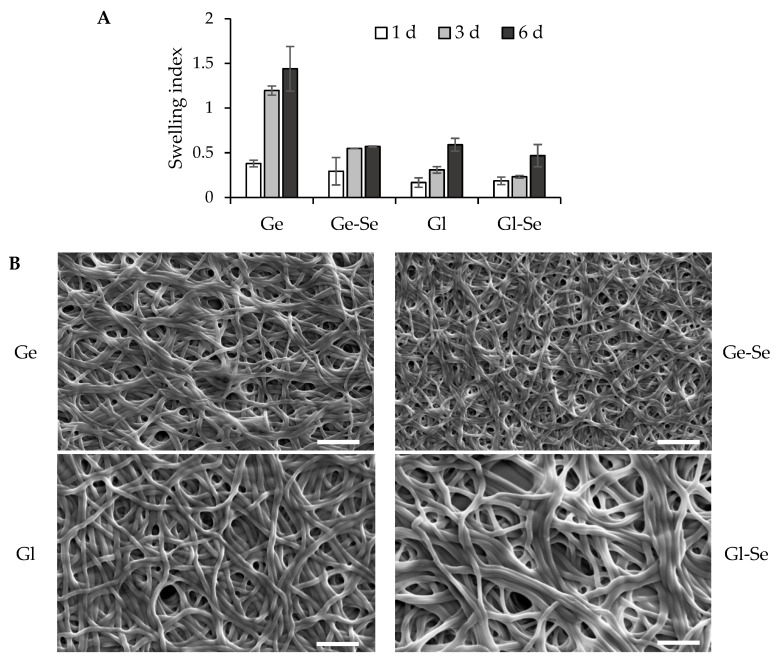
Swelling properties of electrospun scaffolds (**A**) expressed as swelling index (mean values ± s.d.; n = 5). One-way ANOVA; Scheffé test (*p* ≤ 0.05): Ge 3 d vs. Ge-Se 3 d, Gl 3 d, and Gl-Se 3 d; Ge 6 d vs. Ge-Se 6 d, Gl 6 d, and Gl-Se 6 d. SEM images (**B**) of electrospun scaffolds after 6 days of hydration (white line–scale bar: 5 µm).

Figure 6A reports the in vitro swelling behavior of electrospun scaffolds, expressed as swelling index (SI), after 1, 3, and 6 days of incubation in PBS at 37 °C, to evaluate the scaffold’s ability to absorb fluids and preserve its structure after implantation into the lesion. Gelatin-based scaffolds without Se NPs (Ge) show a marked swelling profile, with SI values up to 1.4 after 6 days of hydration. In contrast, gelatin-based scaffolds doped with Se NPs (Ge-Se) have significantly lower swelling capacity, reaching a maximum SI of about 0.5 after 6 days. This reduction could be related to the presence of selenium nanoparticles acting as physical assembly points within the gelatin matrix, which could impair polymer chain mobility and reduce water uptake and matrix relaxation. On the other hand, gliadin-based scaffolds, both undoped (Gl) and doped with Se NPs (Gl-Se), show poor swelling properties, with SI values remaining below 0.6 after 6 days of hydration. This behavior is probably related to the hydrophobicity of gliadin, which is rich in nonpolar amino acid residues. These hydrophobic side chains reduce the protein’s affinity for water, limiting its swelling capacity. In addition, the doping of Se NPs into gliadin fibers does not significantly influence swelling behavior, suggesting that, in this case, the predominant factor in hydration is the hydrophobic nature of the protein matrix. These findings are further supported by SEM analysis (Figure 6B), which shows the morphology of the scaffolds after 6 days of hydration. The images confirm that all scaffolds are capable of absorbing water and of swelling in an aqueous environment, while retaining their fibrous architecture. No significant fiber collapse or fusion is observed, indicating that the scaffolds maintain their structural integrity.

Moreover, contact angle measurements (Figure S4) reveal higher hydrophilicity for the gelatin-based scaffolds compared to the gliadin-based ones. The lower contact angles of the gelatin scaffolds confirm their greater affinity for water, while the higher contact angles of the gliadin scaffolds prove their hydrophobic nature, in agreement with their poor swelling index.

### 3.5. Surface Zeta Potential

**Figure 7:**
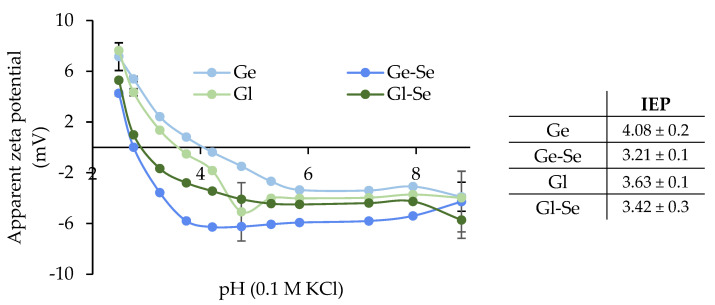
Apparent zeta potential (mV) of the electrospun scaffolds (mean ± s.d.; n = 3). In the inset, the isoelectric points (IEPs) are reported.

The surface zeta potential of the electrospun scaffolds was investigated using surface zeta potential measurements, as shown in Figure 7. All fibers show negative surface charges at alkaline pH, with zeta potential values ranging from approximately −8 to −4 mV. As the pH decreased, all scaffolds displayed a gradual increase in zeta potential, with a sharp transition observed between pH 4 and 5, where the isoelectric point (IEP) was reached. Gliadin typically exhibits an IEP in the range of 6.0–7.0 in its native state [40]; however, in the processed form used for electrospinning, especially when solubilized in hydroalcoholic medium and crosslinked, its apparent IEP is shifted to lower values conceivably due to conformational changes and exposure of acidic residues. Similarly, gelatin, a denatured collagen derivative, generally has an IEP between 4.7 and 5.2, but the lower values observed here suggest partial exposure of acidic groups, such as glutamic/aspartic acid residues. In addition, in both cases, the presence of citric acid as crosslinker could further contribute to a more acidic surface character.

Moreover, the doping with Se NPs leads to a slight decrease in the IEP for both protein-based scaffolds, more markedly in the case of Gl-Se (from 4.08 to 3.21), suggesting that Se NPs interact with positively charged groups of the proteins.

### 3.6. Mechanical Properties

**Figure 8:**
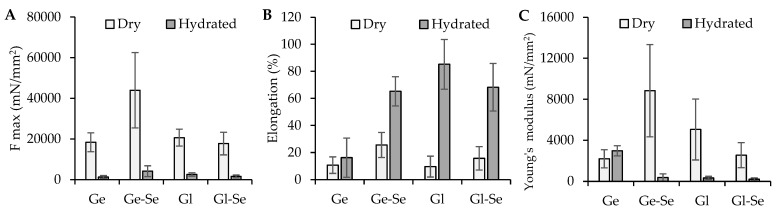
Mechanical properties: (**A**) force at break (mN/mm^2^); (**B**) elongation (%); (**C**) Young’s modulus (mN/mm^2^) (mean ± s.d.; n = 3). One-way ANOVA; Scheffé test (*p* ≤ 0.05): Elongation: Ge hydrated vs. Ge-Se hydrated, Gl hydrated, and Gl-Se hydrated; Young’s modulus: Ge dry vs. Ge-Se dry; Ge hydrated vs. Ge-Se hydrated, Gl hydrated, and Gl-Se hydrated.

The mechanical properties of the electrospun scaffolds were evaluated in both dry and hydrated conditions and expressed as maximum tensile strength (F max), elongation at break, and Young’s modulus (Figure 8). The results reveal substantial differences related to the polymeric matrix (gelatin or gliadin) and the doping with Se NPs.

In dry conditions, gelatin-based scaffolds (Ge and Ge-Se) show higher mechanical resistance compared to gliadin-based ones. In particular, Ge-Se scaffolds have the highest maximum force, suggesting that the doping of Se NPs reinforces the gelatin matrix. On the other hand, gliadin-based scaffolds (Gl and Gl-Se) show lower tensile strength compared to gelatin-based ones, probably due to the reduced chain entanglement typical of gliadin. As expected, hydration leads to a marked drop in tensile strength across all samples.

In contrast, the elongation behavior shows the opposite trend. When hydrated, gliadin-based scaffolds exhibit remarkable flexibility, with elongation values greater than 68%, compared to about 15% in dry conditions. Similarly, gelatin-based fibers became more deformable once hydrated, although to a lesser grade.

The analysis of Young’s modulus further confirms these trends. Dry gelatin scaffolds have the highest stiffness, particularly when Se NPs are present. Conversely, gliadin-based scaffolds remain softer in both dry and hydrated states, with lower moduli reflecting their more elastic nature.

### 3.7. Antioxidant Properties

**Figure 9:**
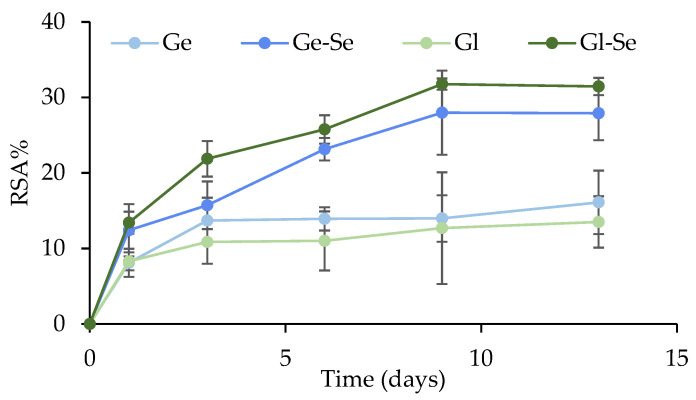
Radical scavenging activity percentage (%) of electrospun scaffolds (mean ± s.d.; n = 3). One-way ANOVA; Scheffé test (*p* ≤ 0.05): Ge 6 d and Gl 6 d vs. Ge-Se 6 d and Gl-Se 6 d; Ge 9 d and Gl 9 d vs. Ge-Se 9 d and Gl-Se 9 d; Ge 12 d and Gl 12 d vs. Ge-Se 12 d and Gl-Se 12 d.

The antioxidant activity of the electrospun scaffolds was measured using the DPPH assay and expressed as radical scavenging activity (RSA%) over a time period of 13 h (Figure 9). Overall, scaffolds show an RSA% increase over time, with differences between Se NP-doped and undoped fibers. In particular, scaffolds containing Se NPs (Ge-Se and Gl-Se) show significantly higher RSA% values compared to their undoped counterparts after 3 h of incubation. This enhanced activity increases up to approximately 8 h, with final RSA% values of about 30%. These profiles are consistent with the presence of Se NPs, as selenium has redox properties capable of scavenging free radicals, thereby attenuating oxidative stress [41,42].

Interestingly, the undoped scaffolds (Ge and Gl) possess detectable antioxidant activity, although the RSA% was lower compared to Se NP-doped scaffolds. This effect could be attributed to the inherent properties of the two proteins. Gelatin and gliadin are both known to contain amino acid residues, such as tyrosine, tryptophan, and cysteine, that can donate electrons or H^+^ to neutralize reactive oxygen species (ROS) [43,44].

As ROS are overproduced during both the inflammatory and proliferative phases of wound healing and have the potential to disrupt tissue regeneration by adversely affecting cells, proteins, and ECM components, the implant of scaffolds characterized by antioxidant properties at the wound bed should facilitate a reduction in oxidative stress, thus stimulating cell proliferation, limiting inflammation, and fostering tissue formation [45]. However, it should be noted that although ROS may be harmful, they play an important physiological role as signaling molecules. In particular, moderate levels of ROS are required for angiogenesis, cell proliferation, and tissue remodeling, while both over-expression and over-suppression could negatively impact the healing cascade. Therefore, a balanced modulation of the wound redox environment is crucial for optimal wound healing [14].

### 3.8. In Vitro Testing

**Figure 10:**
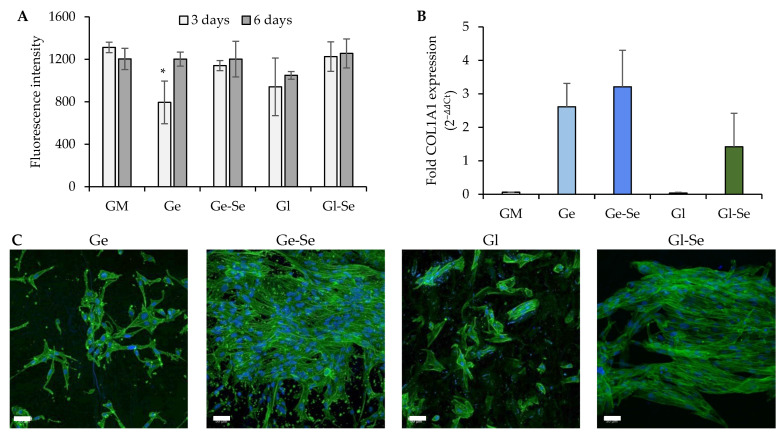
NHDF proliferation (**A**) expressed as fluorescence intensity (FI) after 3 and 6 days of contact with the electrospun scaffolds (mean values ± s.d., n = 5); * indicates significant differences against GM. Relative gene expression level (**B**) of COL1A1 (mean values ± s.d., n = 3); one-way ANOVA; Scheffé test (*p* ≤ 0.05): GM vs. Ge, Ge-Se, and Gl-Se; Gl vs. Ge, Ge-Se, and Gl-Se. CLSM images of NHDFs (**C**) grown onto the electrospun fibers (white bar–scale bar: 50 µm).

Figure 10A shows the cell viability values (fluorescence intensity) of NHDFs after 3 and 6 days of growth onto the electrospun fibers. After 3 days, all scaffolds support cell growth, although the cell growth is lower than that of the control (growth media, GM), which could be explained by the initial adaptation phase that the cells undergo onto the scaffold’s rough topography. After 6 days of growth, a significant increase in fluorescence intensity can be observed for all systems, confirming that the scaffolds are biocompatible and allow cell growth over time.

Moreover, the morphology of NHDFs grown onto the scaffolds after 6 days was evaluated using CLSM (Figure 10C). The images highlight a more pronounced and homogeneous cellular distribution onto the Se NP-doped scaffolds, particularly for Ge-Se, while the undoped counterparts (especially Gl) show less uniform cell adhesion. This observation was further validated by cell quantification, with an average cell density of approximately 2.8 cells/10^3^ µm^2^ for Ge and 4.3 cells/10^3^ µm^2^ for Gl, compared to much higher values of 13.4 cells/10^3^ µm^2^ for Ge-Se and 13.0 cells/10^3^ µm^2^ for Gl-Se. This suggests that the Se NP doping is an important factor that promotes fibroblast adhesion and proliferation.

In addition, quantitative PCR analysis of COL1A1 gene expression (collagen type I, the main ECM component secreted by dermal fibroblasts) supports the trends observed (Figure 10B). The highest degree of gene expression is demonstrated by gelatin-based scaffolds, while gliadin-based scaffolds reveal a lower response, with Gl showing negligible expression. The superior performance in stimulating collagen expression from gelatin-based scaffolds compared to gliadin scaffolds could be related to bioactive gelatin sequences that trigger signal transduction pathways related to cell proliferation and ECM remodeling. In contrast, gliadin exhibits limited bioactive motifs and a higher degree of hydrophobicity, which could impair cell adhesion and spreading. However, in both cases, the presence of Se NPs appears to promote collagen expression, highlighting selenium’s ability to provide a favorable environment for cell proliferation [46].

## 4. Conclusions

In conclusion, electrospun nanofibers based on gelatin and gliadin doped with Se NPs were successfully designed and developed for wound healing. Pullulan was employed as a stabilizing agent, enabling the synthesis of stable, amorphous Se NPs. Pullulan-stabilized Se NPs, blended with either gelatin or gliadin, were then electrospun, leading to the formation of uniform nanofibers with well-distributed nanoparticles within the individual fibers. Physicochemical characterization showed that the influence of the protein source affected fiber morphology, swelling behavior, surface charge, and mechanical properties. Gelatin-based scaffolds had smaller fiber diameters, greater swelling and hydrophilicity capacity, and better mechanical strength, while gliadin-based scaffolds had larger fiber diameters and greater elasticity. Thermal and TEM analyses confirmed the effective doping of Se NPs within the polymeric fibers. Both gelatin and gliadin scaffolds provided negative surface zeta potential at physiological pH and intrinsic antioxidant activity, which increased significantly after doping with Se NP. In vitro testing confirmed scaffold biocompatibility, and, in particular, fibers doped with Se NPs promoted better fibroblast adhesion, proliferation, and ECM-related gene expression than undoped scaffolds. These findings demonstrate that the doping of protein-based fibers with Se NPs boosted the scaffold performance, suggesting a potential benefit in tissue repair.

## Data Availability

The raw data supporting the conclusions of this article will be made available by the authors on request.

## Supplementary Materials

The following supporting information can be downloaded at https://www.mdpi.com/article/10.3390/pharmaceutics17101276/s1: Figure S1: Size distribution of Se NPs (Intensity weighted) prepared in water (black line) or in ethanol/water mixture (grey line); Figure S2: Size distribution of electrospun fibers; Figure S3: Viscosity values (Pa.s) of the polymeric blends at 50, 100, 500 and 100 s-1. The viscosity was measured at 40 °C for Ge and 25°C for Gl polymeric blends (the same conditions used during the electrospinning process) using a rotational rheometer (MCR 102, Anton Paar, Turin, Italy) equipped with a cone plate combination (CP50-1, diameter = 50 mm; angle = 1°); Table S1: Key thermal characteristics of TGA curves; Figure S4. Images of the drop onto the fibers surface after 100 ms. In each image, the value (°) of the contact angle is reported (mean values ± s.d.; n = 3).
