# Supplementary material for: Protein-Based Electrospun Nanofibers Doped with Selenium Nanoparticles for Wound Repair
Source: Pharmaceutics. 2025 Sep 30;17(10):1276. doi: 10.3390/pharmaceutics17101276 (PMC12566627; doi:10.3390/pharmaceutics17101276)
Supplement: Supplementary file 1 [file pharmaceutics-17-01276-s001.zip › pharmaceutics-3847461-supplementary.pdf]

pharmaceutics

Article

Protein-Based Electrospun Nanofibers Doped with Selenium Nanoparticles for Wound Repair

Marco Ruggeri [1], Simone Marsani [1], Amedeo Ungolo [1], Barbara Vigani [1], Eleonora Bianchi [1], Cèsar Viseras [2], Silvia Rossi [1] and Giuseppina Sandri [1,*]

[1] Department of Drug Sciences, University of Pavia, Viale Taramelli 12, 27100 Pavia, Italy; marco.ruggeri@unipv.it (M.R.); simone.marsani01@universitadipavia.it (S.M.); amedeo.ungolo01@universitadipavia.it (A.U.); barbara.vigani@unipv.it (B.V.); elonora.bianchi01@unipv.it (E.B.); silvia.rossi@unipv.it (S.R.)

[2] Department of Pharmacy and Pharmaceutical Technology, University of Granada, Campus of Cartuja, 18071 Granada, Spain; cviseras@unipv.it(C.V.)

* Correspondence: g.sandri@unipv.it(G.S.)

Academic Editor(s): Name

Received: 14 August 2025
Revised: 19 September 2025
Accepted: 27 September 2025
Published: 30 September 2025

Citation: Ruggeri, M.; Marsani, S.; Ungolo, A.; Vigani, B.; Bianchi, E.; Viseras, C.; Rossi, S.; Sandri, G. Protein-Based Electrospun Nanofibers Doped with Selenium Nanoparticles for Wound Repair. *Pharmaceutics* **2025**, *17*, 1276. 10.3390/pharmaceutics17101276

Copyright: © 2025 by the authors. Submitted for possible open access publication under the terms and conditions of the Creative Commons Attribution (CC BY) license (https://creativecommons.org/licenses/by/4.0/).

Supplementary information

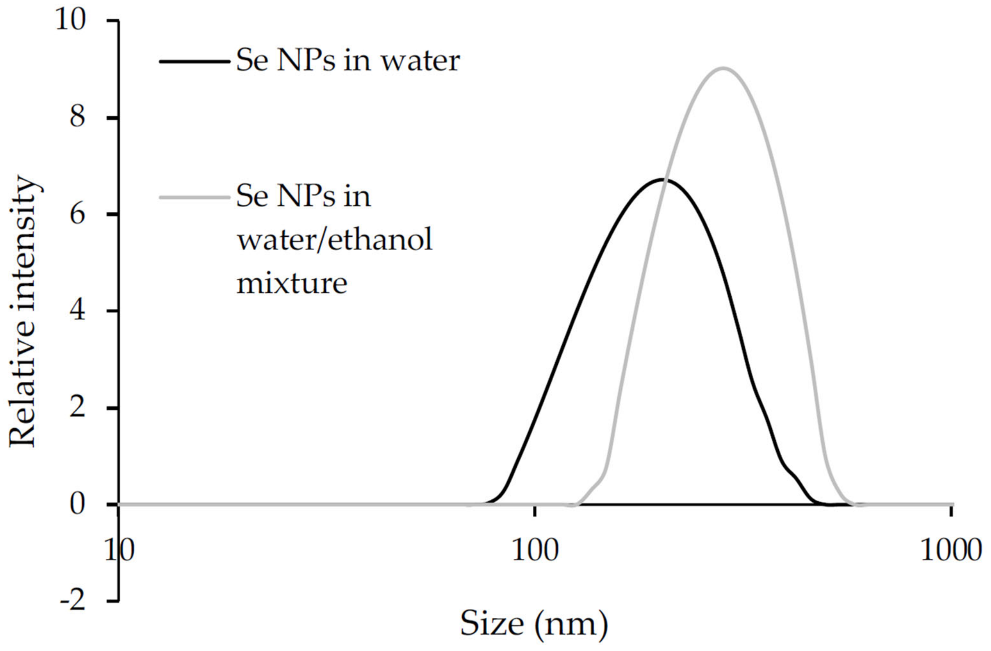

Figure S1. Size distribution of Se NPs (Intensity weighted) prepared in water (black line) or in ethanol/water mixture (grey line).

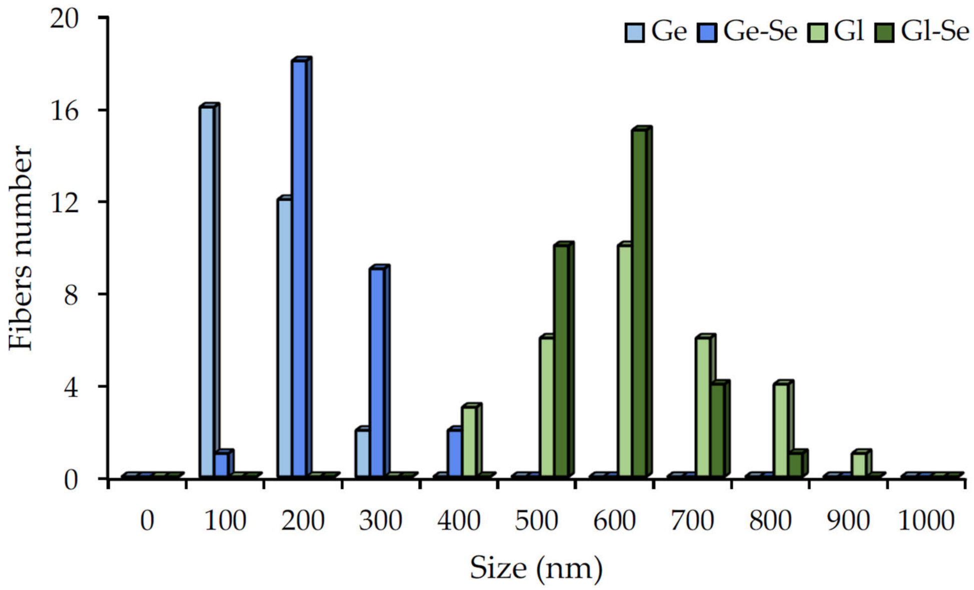

Figure S2. Size distribution of electrospun fibers.

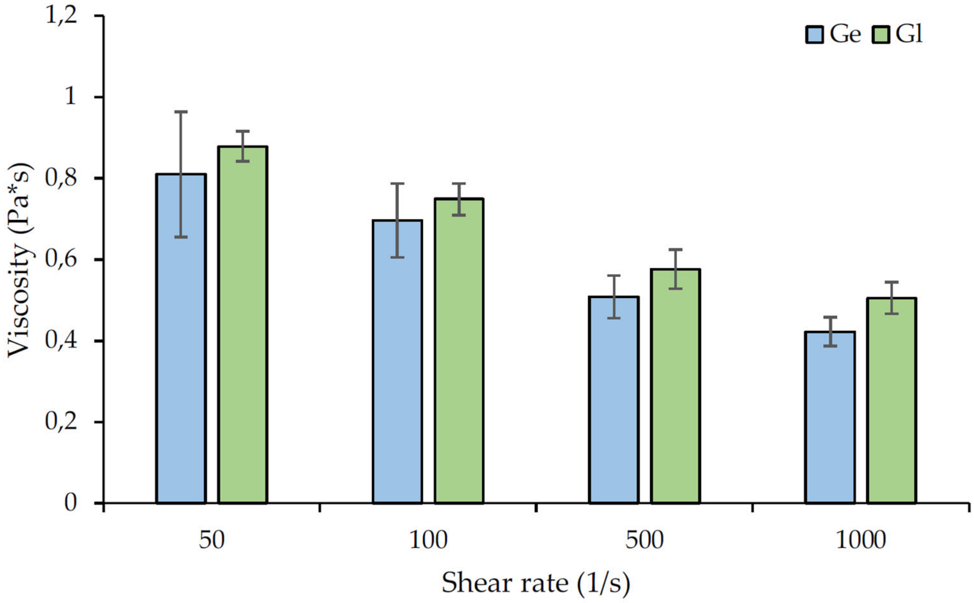

Figure S3. Viscosity values (Pa.s) of the polymeric blends at 50, 100, 500 and 100 s^{-1}. The viscosity was measured at 40°C for Ge and 25°C for Gl polymeric blends (the same conditions used during the electrospinning process)using a rotational rheometer (MCR 102, Anton Paar, Turin, Italy) equipped with a cone plate combination(CP50-1, diameter = 50 mm; angle = 1°).

Table S1. Key thermal characteristics of TGA curves.

Sample	Stage	Δm (%)	Tonset (°C)	Tendset (°C)
Ge	1	7.4	39.9	110.8
	2	67.8	175.9	292.4
	3	24.8	312.7	520.9
GI	1	8.1	41.3	99.7
	2	65.2	164.8	302.0
	3	26.7	310.0	528.2
Ge-Se	1	8.5	42.9	103.8
	2	62.7	165.6	311.9

	3	28.7	324.5	540.2
GI-Se	1	7.7	37.7	121.5
	2	64.1	162.8	298.6
	3	28.1	311.6	544.8

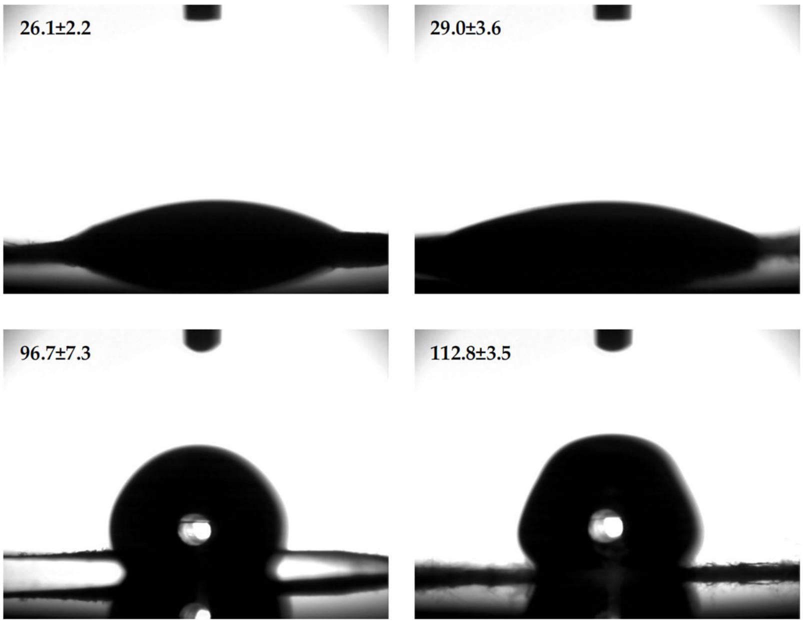

Figure S4. Images of the drop onto the fibers surface after 100 ms. In each image, the value (°) of the contact angle is reported (mean values ± s.d.; n = 3).
